# Study on the piezoresistivity of Cr-doped V_2_O_3_ thin film for MEMS sensor applications

**DOI:** 10.1038/s41378-024-00807-0

**Published:** 2024-12-14

**Authors:** Michiel Gidts, Wei-Fan Hsu, Maria Recaman Payo, Shaswat Kushwaha, Frederik Ceyssens, Dominiek Reynaerts, Jean-Pierre Locquet, Michael Kraft, Chen Wang

**Affiliations:** 1 Micro- and Nanosystems, Department of Electrical Engineering, KU Leuven, Kasteelpark Arenberg 10, 3001 Leuven, Belgium; 2 Functional Oxides Coating Center, Department of Physics and Astronomy, KU Leuven, Celestijnenlaan 200D, 3001 Leuven, Belgium; 3 Manufacturing Processes and Systems, Department of Mechanical Engineering, KU Leuven, Celestijnenlaan 300, 3001 Leuven, Belgium; 4 Flanders Make, Gaston Geenslaan 8, 3001 Leuven, Belgium; 5 Membraanscheidingen, Adsorptie, Katalyse en Spectroscopie voor Duurzame Oplossingen, Department of Microbial and Molecular Systems, KU Leuven, Kasteelpark Arenberg 22, 3001 Leuven, Belgium

**Keywords:** Electrical and electronic engineering, Electronic properties and materials

## Abstract

Cr-doped V_2_O_3_ thin film shows a huge resistivity change with controlled epitaxial strain at room temperature as a result of a gradual Mott metal-insulator phase transition with strain. This novel piezoresistive transduction principle makes Cr-doped V_2_O_3_ thin film an appealing piezoresistive material. To investigate the piezoresistivity of Cr-doped V_2_O_3_ thin film for implementation in MEMS sensor applications, the resistance change of differently orientated Cr-doped V_2_O_3_ thin film piezoresistors with external strain change was measured. With a longitudinal gauge factor of 222 and a transversal gauge factor of 217 at room temperature, isotropic piezoresistivity coefficients were discovered. This results in a significant orientation-independent resistance change with stress for Cr-doped V_2_O_3_ thin film piezoresistors, potentially useful for new sensor applications. To demonstrate the integration of this new piezoresistive material in sensor applications, a micromachined pressure sensor with Cr-doped V_2_O_3_ thin film piezoresistors was designed, fabricated and characterized. At 20 °C, a sensitivity, offset, temperature coefficient of sensitivity and temperature coefficient of offset of 21.81 mV/V/bar, -25.73 mV/V, -0.076 mV/V/bar/°C and 0.182 mV/V/°C, respectively, were measured. This work paves the way for further research on this promising piezoresistive transduction principle for use in MEMS sensor applications.

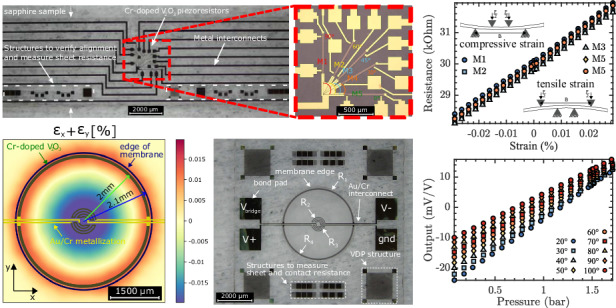

## Introduction

Piezoresistive microelectromechanical systems (MEMS) sensors represent devices that exploit the piezoresistive effect of a material to transduce a stress change, induced by the physical property under observation, into a resistance change. These piezoresistive sensors, such as pressure sensors, accelerometers and strain sensors are frequently used in automotive, aerospace, biomedical and research applications^1,2^. Presently, the majority of piezoresistive sensors utilize doped silicon as piezoresistive material, a material first proposed and employed in the 1950s^3,4^. The success of doped silicon is attributed to its facile integration into MEMS processing technology, cost-effectiveness and robust mechanical bonding to the substrate. However, its piezoresistivity is limited with a longitudinal gauge factor (GF_L_) of 120 or lower, depending on dopant concentration and temperature. Furthermore, only specific metals with a low barrier height, such as Al or Ti, form a good, low Ohmic contact to doped silicon. In the case of lightly doped silicon, which exhibits a higher gauge factor, an additional doping step is often necessary to minimize contact resistivity^5^. The pursuit of enhanced performance, reduced power consumption, increased robustness and stability for piezoresistive MEMS sensors drives researchers to explore new piezoresistive materials^1,6,7^.

An intriguing category of materials for use in piezoresistive applications is vanadium oxide materials. Various studies in literature demonstrated the piezoresistive properties of different phases of vanadium oxide materials. For instance, in ref. ^8^, a strain sensor was constructed using a VO_2_ nanobeam as the piezoresistive material. The piezoresistivity and phase of the VO_2_ nanobeam were examined through electrical characterization and Raman spectrum measurements. A gauge factor (GF) in the range of 225 - 347 was reported, likely resulting from a monoclinic M1-M2 phase transition, as confirmed by Raman spectroscopy, which indicates that strain adjusted the fraction of M1 and M2 phase. However, an observed hysteresis effect when stressing the sample makes the material less suitable for sensor applications. Similarly, VO_2_ nanowires subjected to uniaxial tensile strain at room temperature yielded a GF of 51^9^. Nevertheless, these nanobeams and nanowires pose challenges for integration into MEMS sensor processing. Thin film (TF) materials are much more interesting materials for integration in piezoresistive sensor applications. In ref. ^10^, a pressure sensor utilizing a piezoresistive vanadium oxide thin film as the sensing material was demonstrated. The piezoresistors, with a thickness of 370 nm, were deposited using radio frequency magnetron sputtering on SiO_2_. X-ray photoelectron spectroscopy revealed that the composition of the deposited VO_x_ TF consisted of 25% VO_2_ and 75% V_2_ O_5_. Despite a measured GF of 259, no explanation for this substantial piezoresistivity was provided. Additionally, no measurements of resistance change with temperature or stress orientation resistance change measurements were conducted. This makes it unclear how good the performance of a MEMS sensor would be with integrated VO_x_ TF piezoresistors from ref. ^10^.

A promising vanadium oxide TF material is Cr-doped V_2_O_3_. With application of hydrostatic pressure, Cr doping or non-stoichiometry, bulk V_2_O_3_ has got a rich phase diagram with a paramagnetic metal (PM) - paramagnetic insulating (PI) phase transition at room temperature^11–13^. This phase transition is of interest for piezoresistive applications due to its significant resistivity change. For sputtered Cr-doped V_2_O_3_ TF, the phase can be stabilized under stress^14^. Similar observations were made for molecular beam epitaxy (MBE) grown single crystal Cr-doped V_2_O_3_ TF in refs. ^15^ and ^16^. No abrupt resistivity change with temperature caused by a PM-PI phase transition was measured for Cr-doped V_2_O_3_ TF of different Cr doping levels, internal strain hinders the phase transition with temperature. Instead, a gradual change in room temperature resistivity (RTR) from PM to PI with increasing Cr doping level was observed, indicating an intermediate phase region, phase coexistence of PM and PI regions as seen in refs. ^17^ and^18^. Interestingly, strain can induce the Mott metal-insulator transition (MIT) at room temperature in both pure and Cr-doped compounds. Pia et al. demonstrated this with strain engineering the in-plane lattice parameters between 4.943 Å and 5.037 Å by varying the precise composition of the thin film buffer layer in ref. ^19^. It was shown that the effective transfer of strain achieved by heteroepitaxy (internal) strain leads to a Mott MIT in a wide range of temperatures around room temperature and a substantial resistivity change. It is anticipated that a similar Mott MIT can be induced with external strain. To investigate this, Cr-doped V_2_O_3_ TFs with 4% Cr doping were fabricated with MBE and strained with four-point bending. By varying the Cr doping level, the resistivity and resistivity change with temperature of the material can be varied^16^. The impact of the doping level on the piezoresistivity is determined by the impact of the doping level on the in-plane lattice parameter of the thin film material and the percentage of the PM and PI phase regions in the material. For a doping level of 4%, the in-plane lattice parameter is around 4.98 Å, this was previously identified as approximately in the middle of the PM-PI transition for internal strained Cr-doped V_2_O_3_ TFs^19^. The expectation is that for the middle of the PM-PI transition, with a similar percentage of PM and PI phase regions, the phase transition sensitivity with strain is maximized. The stabilization of the Mott PM-PI transition with temperature around room temperature and gradual phase transition from metallic to insulating with strain makes Cr-doped V_2_O_3_ TF an ideal piezoresistive material. To demonstrate the integration of this new piezoresistive material in sensing application, a MEMS pressure sensor with Cr-doped V_2_O_3_ TF piezoresistors was designed, fabricated and characterized.

Cr-doped V_2_O_3_ TF in the PM, PI phase and intermediate phase is a trigonal (rhombohedral) crystal belonging to the $\bar{3}{\rm{m}}$ crystal class and space group: ${\rm{R}}\bar{3}{\rm{c}}$. Notice that the PI phase is isostructural with respect to the PM phase. The piezoresistivity coefficient tensor is given by^20^:

1 $${\pi }_{{Cr}-{V}_{2}{O}_{3}}=\left[\begin{array}{cccccc}{\pi }_{11} & {\pi }_{12} & {\pi }_{13} & {\pi }_{14} & 0 & 0\\ {\pi }_{12} & {\pi }_{11} & {\pi }_{13} & {-\pi }_{14} & 0 & 0\\ {\pi }_{31} & {\pi }_{31} & {\pi }_{33} & 0 & 0 & 0\\ {\pi }_{41} & -{\pi }_{41} & 0 & {\pi }_{44} & 0 & 0\\ 0 & 0 & 0 & 0 & {\pi }_{44} & 2{\pi }_{41}\\ 0 & 0 & 0 & 0 & {\pi }_{14} & {\pi }_{11}-{\pi }_{12}\end{array}\right]$$

The longitudinal, transversal and shear piezoresistivity coefficients are given by ${\pi }_{L}={\pi }_{11}$, ${\pi }_{T}={\pi }_{12}$ and ${\pi }_{S}=0$. When a piezoresistor is rotated to the resistor axis system (in-plane, as shown in Fig. S1 of the Supplementary Material), the piezoresistivity coefficient π_L_, π_T_ and π_S_ in the resistor axis system can be expressed as:

2 $${\pi }_{L}={\pi }_{11}^{{\prime} }={\pi }_{11}$$

3 $${\pi }_{T}={\pi }_{12}^{{\prime} }={\pi }_{12}$$

4 $${\pi }_{S}={\pi }_{16}^{{\prime} }=0$$

Analogous to in-plane piezoresistors fabricated on (111) silicon, the longitudinal, transversal and shear piezoresistivity coefficients of Cr-doped V_2_O_3_ are independent of the rotation angle φ of the piezoresistor within the XY plane of the crystallographic framework. The mathematical derivation of this can be found in the Supplementary Material. Upon application of stress ${{\rm{\sigma }}}^{{\prime} {\prime} }$ to a Cr-doped V_2_O_3_ TF piezoresistor orientated at an angle φ + θ relative to the principle stress axis system (see Supplementary Material Fig. S1), the resistance change is given by:

5 $$\begin{array}{c}\frac{\Delta R}{R}\left(\varphi ,\theta \right)={\pi }_{L}\Delta {\sigma }_{L}+{\pi }_{T}\Delta {\sigma }_{T}+{\pi }_{S}\Delta {\sigma }_{S}\\ \begin{array}{c}=({\pi }_{11}{\cos }^{2}\left(\varphi +\theta \right)+{\pi }_{12}{\sin }^{2}(\varphi +\theta ))\Delta {\sigma }_{x}^{{\prime} {\prime} }\\ +({\pi }_{11}{\sin }^{2}\left(\varphi +\theta \right)+{\pi }_{12}{\cos }^{2}(\varphi +\theta ))\Delta {\sigma }_{y}^{{\prime} {\prime} }\end{array}\\ +({\pi }_{11}-{\pi }_{12})\sin (2(\varphi +\theta ))\Delta {\sigma }_{{xy}}^{{\prime} {\prime} }\end{array}$$

The variation in resistance with stress of a Cr-doped V_2_O_3_ TF piezoresistor is exclusively determined by the piezoresistivity coefficients π_11_ and π_12_ in conjunction with the angular orientation φ + θ of the piezoresistor relative to the principle stress axis system. The longitudinal and transversal GF are defined by:

6 $${{GF}}_{L}=\frac{\frac{\Delta R}{R}\left(\varphi +\theta =0^{\circ} \right)}{\varepsilon }=\frac{{\pi }_{11}\Delta {\sigma }_{x}^{{\prime} {\prime} }+{\pi }_{12}\Delta {\sigma }_{y}^{{\prime} {\prime} }}{\varepsilon }$$

7 $${{GF}}_{T}=\frac{\frac{\Delta R}{R}\left(\varphi +\theta =90^{\circ} \right)}{\varepsilon }=\frac{{\pi }_{12}\Delta {\sigma }_{x}^{{\prime} {\prime} }+{\pi }_{11}\Delta {\sigma }_{y}^{{\prime} {\prime} }}{\varepsilon }$$

## Materials and methods

### Thin film growth, structural characterization and sample fabrication

**Fig. 1:**
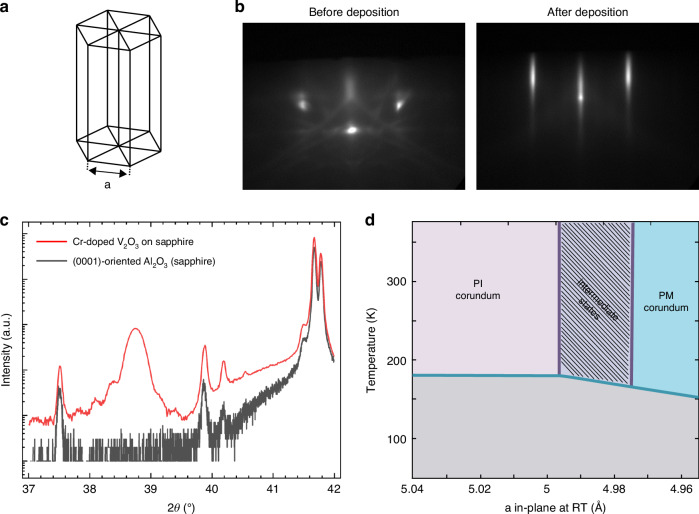
Characterisation of Cr-doped V_2_O_3_ TF. **a** Corundum crystal structure (space group: ${\rm{R}}\bar{3}{\rm{c}}$) with a the in-plane lattice parameter. **b** RHEED image taken at room T before and after deposition. **c** XRD *ϕ* scan. **d** Phase diagram of the ${({{\rm{V}}}_{1-{\rm{x}}}{{\rm{M}}}_{{\rm{x}}})}_{2}{{\rm{O}}}_{3}$ system for thin films from ref. ^19^

Cr-doped V_2_O_3_ TFs, intended for piezoresistive analysis, were deposited on $\left(0001\right)-{{\rm{Al}}}_{2}{{\rm{O}}}_{3}$ (sapphire) substrates by oxygen-assisted MBE. The MBE chamber maintained a base pressure of 10^−9^ mbar and was equipped with Reflection High Electron Energy Diffraction (RHEED) apparatus to verify the epitaxial growth in situ. A chromium concentration of 4% was achieved by co-deposition of vanadium and chromium metals in an O_2_ partial pressure of 8.2–8.5 × 10^−6^ Torr. Vanadium was evaporated from an electron gun with a deposition rate of 0.1 Å/s, chromium was evaporated from a Knudsen cell (Veeco) with the rate of 0.04 Å/s. The flux of the evaporated material was calibrated by the quartz crystal microbalance before and after thin film growth to check the stability. The crystalline structure of the as-grown films were ascertained via high-resolution X-ray diffraction (XRD) with a Panalytical X’pert Pro diffractometer using a copper anode. The excellent structural quality is evidenced by the presence of finite-size oscillations in out-of-plane $2{\rm{\theta }}-{\rm{\omega }}$ XRD measurements (see Fig. 1c). RHEED analyses conducted pre- and post-deposition (illustrated in Fig. 1b) provided insights into the morphological stucture of the films, with post-deposition RHEED imagery revealing distinct streak patterns, indicates the thin film is flat and epitaxially grown on the sapphire substrate.

**Fig. 2:**
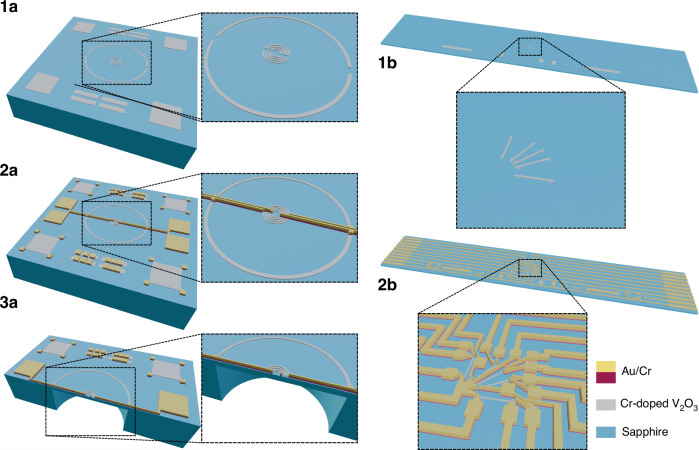
Fabrication steps. Illustration of the fabrication steps of Cr-doped V_2_O_3_ TF pressure sensor and bending sample. **1a**, **1b** Cr-doped V_2_O_3_ TF deposition and etching on sapphire substrate. **2a**, **2b** Deposition and lift-off of Cr and Au metal contacts. **3a** Milling of sapphire to fabricate sapphire membrane

Subsequent to the Cr-doped V_2_O_3_ TF deposition, piezoresistors of 500 μm long and 25 μm wide were fabricated by etching the TF with a SF_6_ + O_2_ rich plasma reactive ion etching process. Additionally, an aqua regia or hydrofluoric acid solution was also identified to effectively etch the material. Contacts to the metal were established with deposition of chromium and gold via magnetron sputtering, facilitated by a lift-off process as illustrated in Fig. 2. Finally, the sapphire substrate was diced in rectangular beams, measuring 38 mm in length and 5.3 mm in width. Figure 2 provides a detailed view of the fabrication process of the Cr-doped V_2_O_3_ piezoresistors on the sample. The Cr-doped V_2_O_3_ TF piezoresistors have a measured thickness by XRR of 66 nm and sheet resistance of 1305 Ohm/sq measured with the van der Pauw method.

### Electrical and mechanical characterization

**Fig. 3:**
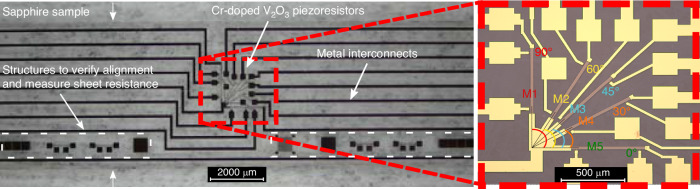
Cr-doped V_2_O_3_ TF piezoresistors. Picture of fabricated Cr-doped V_2_O_3_ TF piezoresistors on a sapphire beam sample with close-up of the differently orientated piezoresistors

To determine the longitudinal and transversal GF of Cr-doped V_2_O_3_ TF, the resistance change with strain of differently orientated Cr-doped V_2_O_3_ TF piezoresistors was measured. One with its long direction parallel to the applied strain ($\varphi +\theta =0^{\circ}$) and one perpendicular to the strain ($\varphi +\theta =90^{\circ}$), see Formula 6 and 7. The resistance change of three additional piezoresistors with different orientation ($\varphi +\theta ={30^{\circ}}, {45^{\circ}}, 60^{\circ}$) was measured to verify the result (see Fig. 3). To create a uniform strain, the piezoresistors were fabricated on a thin beamlike sapphire sample and bended with a four-point bending test setup.

The four-point bending test setup was composed of an aluminum framework, components produced via three-dimensional printing and various electrical elements (see Supplementary Material Fig. S2). To quantify the strain elicited in the sample and the piezoresistors by this applied force, simulations were conducted using the COMSOL Multiphysics software, see Supplementary Material Fig. S3. The simulated strain come close to the theoretical maximum strain derived with Euler–Bernoulli beam theory. With *W* the width of the sample, *t* the thickness of the sample, *E* the Young’s modulus and *B* the distance between the outer beams, the maximum strain in the sample is given by^21^:

8 $${\varepsilon }_{x,\max }^{{\prime} {\prime} }=\frac{3{FB}}{4W{t}^{2}E}$$

Prior to employing the four-point bending setup for evaluating the piezoresistive properties of Cr-doped V_2_O_3_ TF piezoresistors, the experimental setup underwent a rigorous validation process. This validation involved monitoring the resistance change in a doped silicon piezoresistor, dimensions 500 μm by 25 μm by 2.12 μm, embedded within a silicon test sample, and correlating the observed changes with theoretical predictions. The silicon test sample was fabricated from a silicon-on-insulator wafer, with the top layer doped to a uniform boron doping level. The piezoresistor were fabricated utilizing deep reactive ion etching (DRIE), followed by aluminum metallization through magnetron sputtering and subsequent lift-off process. A section of the wafer, measuring 38 mm in length and 5.4 mm in width, was diced from the wafer and its thickness was precisely measured to be 467 μm with a tolerance of ±1 μm. The orientation of the piezoresistor was meticulously aligned along the [110] crystallographic direction to within 0.15° using additional crystal alignment identification steps in the fabrication process, ensuring its central placement within the sample. The bending tests were conducted under strictly controlled climatic conditions within the laboratory, with continuous temperature monitoring. To mitigate any external vibrational interference, the measurements were performed atop a vibration isolation table.

The electrical resistivity of the piezoresistor was determined through van der Pauw methodology, yielding a value of 0.0484 Ohm-cm. This measurement facilitated the calculation of the doping concentration, which was found to be $N=7.036{e}^{17}{{\rm{cm}}}^{-3}$. At the specific testing temperature of 19.9 °C, the piezoresistive coefficient, P(N,T), was computed using Richter’s model to be 0.855^22^. The resistance change of the doped silicon piezoresistor is given by:

9 $$\frac{\Delta R}{R}\left(\varphi ={45^{\circ}} ,\theta =45^{\circ} \right)=\frac{1}{2}0.855(\pi _{11,{si}}\left({N}_{0},{T}_{0}\right)+{\pi }_{12,{si}}\left({N}_{0},{T}_{0}\right)+{\pi }_{44,{si}}\left({N}_{0},{T}_{0}\right))\Delta {\sigma }_{x}^{{\prime} {\prime} }$$

**Fig. 4:**
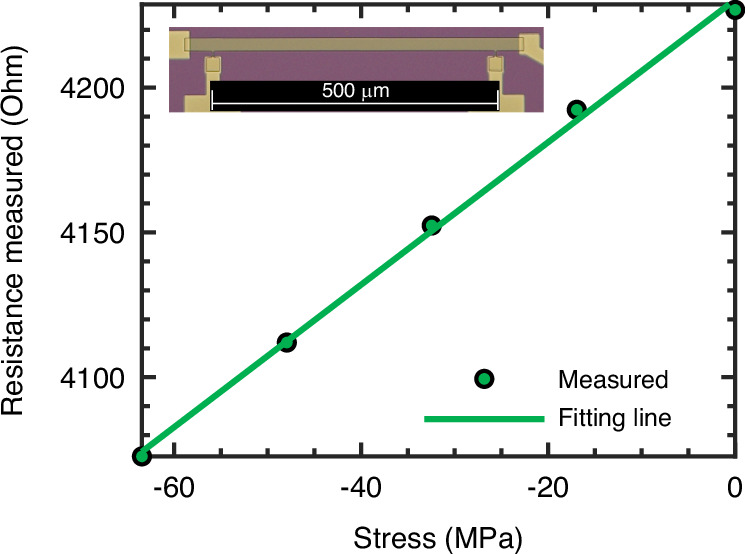
Doped Si resistance change with stress change. Measured resistance change with stress change for a doped silicon piezoresistor stressed by four-point bending

With ${{\rm{\pi }}}_{11,{\rm{si}}}\left({{\rm{N}}}_{0},{{\rm{T}}}_{0}\right)$, ${{\rm{\pi }}}_{12,{\rm{si}}}\left({{\rm{N}}}_{0},{{\rm{T}}}_{0}\right)$ and ${{\rm{\pi }}}_{44,{\rm{si}}}\left({{\rm{N}}}_{0},{{\rm{T}}}_{0}\right)$ the piezoresistivity coefficients for moderately doped (100) silicon^23^. The stress change ${\Delta {\rm{\sigma }}}_{{\rm{x}}}^{{\prime} {\prime} }$ was simulated using COMSOL. For the experiment the sample was bended downwards, resulting in an increasing compressive stress change. The resistance was measured using a Keithley 2450 sourcemeter by forcing a current of 5 μA through the piezoresistor and measuring the voltage (four-point probing method). In Fig. 4 the resistance of the piezoresistor is plotted as a function of the stress. For each stress, 75 resistance measurement points were taken, the median of these points is shown. The measured resistance change of **–**2.46 Ω/MPa is very similar to the expected simulated resistance change of **–**2.59 Ω/MPa.

Before straining the Cr-doped V_2_O_3_ TF piezoresistors, an initial evaluation was performed to establish the linearity of the current-voltage (I-V) relationship. This preliminary step involved modulating the current across each piezoresistor while concurrently recording the resultant voltage changes to affirm a linear I-V correlation. For the flexural tests, a constant current of 50 μA was directed through the piezoresistors, and the resultant voltage was accurately measured employing a Keithley 2450 instrument, adhering to the four-point probe methodology. The median value derived from a series of ten measurements per resistor was used for analysis. The flexural deformation of the sample was incrementally increased by methodically adding weights to the experimental setup, with the resistance of the piezoresistors being meticulously recorded at each stage of deformation. The resistance measurements were also repeated during the gradual removal of the weights to evaluate any potential hysteresis. Following the completion of the tensile flexural testing, the sample was detached from the apparatus, and the positions of the 3D-printed beam structures at the base and top were interchanged to introduce compressive strain within the piezoresistors for subsequent compressive flexural testing, maintaining the same procedural rigor as in the tensile testing phase. Throughout the entirety of the experimental procedure, the four-point bending apparatus was stationed on a vibration-damping table to negate the influence of extraneous vibrations. Ambient temperature was continuously monitored using an external temperature sensor, with a consistent temperature of 19.9 °C being recorded. Additionally, to investigate the resistance change with temperature, the resistance of a M5 piezoresistor was measured at varying temperatures utilizing a probe station equipped with a temperature-controlled chuck.

### Sensor fabrication, design and characterization

For the fabrication of the Cr-doped V_2_O_3_ TF MEMS pressure sensor, the same process as for the Cr-doped V_2_O_3_ TF piezoresistors was followed with the addition of a milling process at the end to fabricate a membrane. First the piezoresistors were fabricated with MBE, etched with RIE and Cr/Au was sputtered as interconnect material. After this the membrane was fabricated by diamond grinding and milling on Kern MMP micro-milling center. The final membrane had a 4.2 mm diameter and a measured thickness of 98 μm.

Since the longitudinal and transversal piezoresistivity coefficient of Cr-doped V_2_O_3_ TF piezoresistors were found to be equal in sign $({\pi }_{11}\approx {\pi }_{12})$, see Section Discussion, it is impossible to make a full Wheatstone bridge with four Cr-doped V_2_O_3_ TF piezoresistors on the side of the membrane as is common for pressure sensor with doped silicon piezoresistors^4^. To make a Wheatstone bridge with Cr-doped V_2_O_3_ TF piezoresistors, two piezoresistors were placed in the middle of the membrane, and two were placed on the side of the membrane. Both in the region of the biggest strain change with pressure. A simulation of the strain *ε*_*x*_ + *ε*_*y*_ on top of the sapphire membrane was done to find the most ideal location of the piezoresistors (pressure of 2 bar, 100 μm thick membrane, 4.2 mm membrane diameter). The sheet resistance of Cr-doped V_2_O_3_ TF is, depending on the doping level in silicon, roughly estimated a factor of 2 to 100 times bigger than doped silicon piezoresistors^5^, this allows to place the piezoresistor more in concentrated stress areas compared to doped silicon, increasing sensitivity.

The characterization of the pressure sensor was conducted across a spectrum of pressures and temperatures utilizing the apparatus slightly modified from ref. ^24^, depicted in Fig. S4 from the Supplementary material. The measurement setup ensures systematic incrementation of differential pressure from 0 to 1.8 bar in 0.1 bar increments at each designated temperature, with the procedure being replicated multiple times to ensure reliability.

## Results

### Cr-doped *V*_2_*O*_3_ TF piezoresistors

**Fig. 5:**
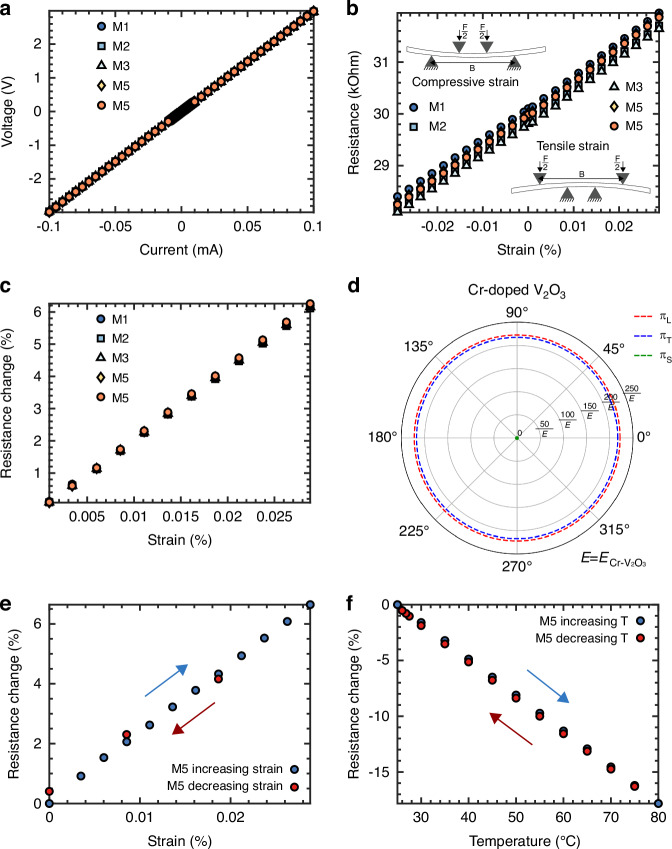
Cr-doped V_2_O_3_ TF electrical and stress measurements. **a** I-V curve of Cr-doped V_2_O_3_ TF piezoresistors. **b** Resistance of Cr-doped V_2_O_3_ TF piezoresistors with applied strain. **c** Resistance change of Cr-doped V_2_O_3_ TF piezoresistors with positive applied strain. **d** Cr-doped V_2_O_3_ TF piezoresistivity coefficients for different orientations of the piezoresistor in the XY plane of the crystallographic framework. **e** Resistance change of Cr-doped V_2_O_3_ TF piezoresistor M5 with increasing and decreasing strain. **f** Resistance change of Cr-doped V_2_O_3_ TF piezoresistor M5 with increasing and decreasing temperature

The fabricated sample is shown in Fig. 3. Illustrated in Fig. 5a is the current-voltage (I-V) characteristic of Cr-doped V_2_O_3_ TF piezoresistors, alongside the resistance variation under applied strain for the differently orientated piezoresistors. The I-V profile for these Cr-doped V_2_O_3_ TF piezoresistors demonstrates a linear relation between the current through the piezoresistors and the resultant voltage across them, signifying an absence of notable self-heating within these thin film piezoresistive components and good electrical contact between the piezoresistors and the chromium metal. As depicted in Fig. 5b–d, the Cr-doped V_2_O_3_ TF piezoresistors exhibit significant piezoresistivity for all orientations, with a notable resistance change of 665 Ohm (2.2%) upon the application of 0.01% strain change. A marginal reduction in piezoresistivity under negative strain was noted, which is likely attributable to aspects related to the measurement setup. Furthermore, for piezoresistor M5 also the resistance change with decreasing strain was measured as shown in Fig. 5e. While a minor discrepancy from the resistance trend observed with increasing strain was noted, no definitive hysteresis effect was evident. From the resistance change with temperature measured (see Fig. 5f), no pronounced hysteresis or abrupt resistance change was detected across a temperature range from 28 °C to 80 °C. This observation underscores the stability of the Cr-doped V_2_O_3_ TF in its intermediate phase. The temperature coefficient of resistivity (TCR) measured at 28 °C was 0.3204%/°C, which is comparatively lower than the TCR values reported for p-type doped silicon with doping concentrations below 10^17^ cm^−3^ ^1^.

With longitudinal and transverse gauge factors of *GF*_*L*_ = 222 and *GF*_*T*_ = 217, Cr-doped V_2_O_3_ TF piezoresistors are more piezoresistive than doped silicon. The reported longitudinal gauge factor aligns closely with the piezoresistive characteristics reported for other vanadium oxide materials^8–10^. Unexpectedly, the same resistance change with strain was observed for all the piezoresistors despite being orientated with their long direction in a different direction to the applied strain. Since the GF_L_ and GF_T_ are approximately the same, also π_11_ and π_12_ are approximately the same. With *π*_11_ *=* *π*_12_, the resistance change for Cr-doped V_2_O_3_ TF piezoresistors is given by:

10 $$\frac{\Delta R}{R}\left(\varphi ,\theta \right)=\frac{\Delta R}{R}={\pi }_{11}\left(\Delta {\sigma }_{x}^{{\prime} {\prime} }+\Delta {\sigma }_{y}^{{\prime} {\prime} }\right)$$

### MEMS Cr-doped *V*_2_*O*_3_ TF pressure sensor

**Fig. 6:**
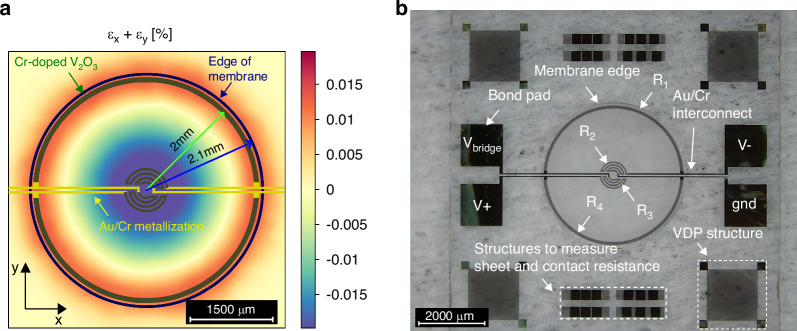
Cr-doped V_2_O_3_ sapphire pressure sensor. **a** Simulated strain *ε*_*x*_ + *ε*_*y*_ on the membrane for a pressure of 2 bar with superimposed piezoresistors and metallization for visualization. **b** Picture of fabricated Cr-doped V_2_O_3_ TF piezoresistors on a sapphire beam sample with close-up of the differently orientated piezoresistors

**Fig. 7:**
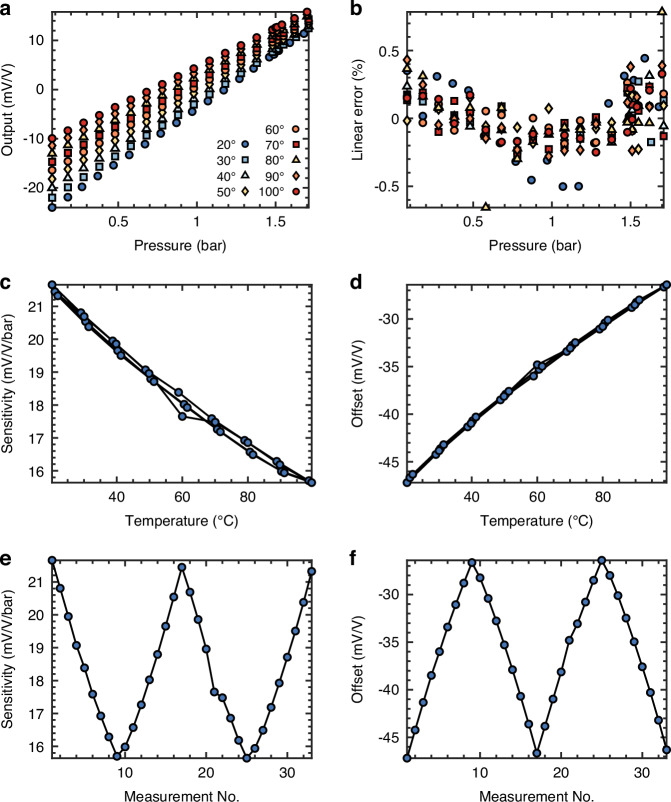
Cr-doped V_2_O_3_ sapphire pressure sensor measurement result. **a** Measured output voltage in mV/V of the Wheatstone bridge. **b** Linearity error in % span with different temperatures and pressures. **c** Repeated sensitivity measurements at different temperatures. **d** Repeated offset measurements at different temperatures. **e** Measured sensitivity when cycling the pressure and temperature. **f** Measured offset when cycling the pressure and temperature

The fabricated pressure sensor is shown in Fig. 6b. The Wheatstone bridge output voltage of the sapphire pressure sensor with Cr-doped V_2_O_3_ TF piezoresistors is depicted in Fig. 7a, showing its dependency on applied pressure and temperature. A linear relationship is evident between the output voltage and applied pressure. At 20 °C, a sensitivity of 21.81 mV/V/bar, an offset of -25.73 mV/V, a temperature coefficient of sensitivity of -0.076 mV/V/bar/°C and a temperature coefficient of offset of 0.182 mV/V/°C were measured. The observed offset at all temperatures is mostly attributed to fabrication mismatch of resistance between the piezoresistors. The substantial sensitivity of the pressure sensor is due to the high piezoresistivity of Cr-doped V_2_O_3_ TF, a large membrane and the contribution of both ε_x_ and ε_y_ to the total resistance change of the piezoresistors. In Fig. 6a, the piezoresistors and metallization are superimposed on the *ε*_*x*_ + *ε*_*y*_ strain simulation to show their placement with respect to the membrane edge. Notice that the inner piezoresistors decrease bigger in resistance than the outer piezoresistors increase as they see a bigger contribution of *ε*_*x*_ + *ε*_*y*_. The mismatch in resistance change between the inner and outer piezoresistors leads to increased sensor nonlinearity (see Fig. 7b), which can be mitigated by adjusting the placement of the inner piezoresistors at the expense of reduced sensitivity. As temperature increases, the sensitivity and offset decrease due to the reduced piezoresistivity of Cr-doped V_2_O_3_ TF. However, no abrupt resistance change of the piezoresistors was observed, indicating successful stabilization of the intermediate phase and gradual phase transition with strain, consistent with previous four-point bending strain measurements of Cr-doped V_2_O_3_ TF piezoresistors. Repeated pressure and temperature cycles demonstrate negligible hysteresis, rendering Cr-doped V_2_O_3_ TF pressure sensors suitable for straightforward sensor calibration (see Fig. 7c–f).

## Discussion

### Isotropic piezoresistivity coefficients

The unique property of isotropic piezoresistivity coefficients (*π*_11_ = *π*_12_) was previously reported for materials such as annealed copper, copper-nickel alloys^25^ and ytterbium^26^. It is noteworthy to differentiate this phenomenon from the same term (“isotropic piezoresistance”) occasionally used in literature, referring to the piezoresistivity coefficients being invariant with respect to crystal orientation^27^. However, the equality of *π*_11_ = *π*_12_ is distinct and not typically anticipated in materials with piezoresistivity coefficients independent of the in-plane rotation angle of the piezoresistor with respect to crystal orientation, such as in-plane piezoresistors fabricated on (111) silicon, polycrystalline piezoresistors or p-type 4H-SiC piezoresistors in the (0001) plane. For the copper-nickel alloys of ref. ^25^, the measured GFs are roughly 300 times smaller than what is measured for Cr-doped V_2_O_3_ TF (assuming a Young’s modulus ${E}_{{cu}}=130\,{\rm{GPa}}$). In ref. ^26^, piezoresistivity coefficients of ${\pi }_{11,{YT}}=-20{e}^{-11}{{\rm{Pa}}}^{-1}$ and ${\pi }_{12,{YT}}=-19{e}^{-11}{{\rm{Pa}}}^{-1}$ were measured, resulting in small GFs: ${{GF}}_{L}=-3.74$ and ${{GF}}_{T}=-3.553$ for *E*_*YT*_ = 187 kbar. The noteworthy isotropic piezoresistivity of Cr-doped V_2_O_3_ TF, with such significant GFs, has not been previously reported for piezoresistive materials, presenting novel avenues for application in sensor technology. Specifically, in strain gauge applications, this property negates the necessity for precise alignment of the gauge with the strain direction, a requirement in conventional doped silicon or metal strain gauges to avoid measurement errors. However, a limitation of Cr-doped V_2_O_3_ TF piezoresistors is their inability to measure σ_x_ and σ_y_ separately.

Prior investigations posited that Cr-doped V_2_O_3_ TF occupy an intermediary phase between the PM and PI phase, with indications of phase coexistence at the nanoscale (see Fig. 1d)^17–19^. The piezoresistive analyses conducted in the present study lend empirical support to the hypothesis of such phase coexisting domains within these thin films. Upon the application of incremental strain, discrete nanoscale domains with the PM phase are subjected to critical distortions of their in-plane lattice parameters, precipitating an abrupt phase transition into the PI state (local Mott MIT). This localized transition contributes to a more global observed gradual resistance change across the entire piezoresistive element. Notably, these phase transitions appear reversible, as evidenced by the restoration of initial resistive properties upon the alleviation of strain (see Fig. 5e), a phenomenon also documented previously in literature^17^. The observed isotropy in piezoresistivity coefficients of Cr-doped V_2_O_3_ TFs may be attributable to these localized phase transitions. Given that the piezoresistors labeled M1 through M5 were fabricated from identical Cr-doped V_2_O_3_ TF material, they encompass a comparably homogeneous distribution of nanoscale domains in the PM to PI phase. This uniform distribution ensures that, in their unstressed state, all piezoresistors exhibit a similar ratio of PM-PI domains and thus a similar resistance, predicated on the assumption of a substantial quantity of such nanoscale domains. Moreover, a uniform strain across differently oriented piezoresistors induces phase transitions in a proportionate number of nanoscale domains for each piezoresistor, owing to the orientation-independent nature of the piezoresistivity coefficients of Cr-doped V_2_O_3_ TF with respect to the crystal orientation. Consequently, under strain, the differently orientated piezoresistors see a similar change in ratio of nanoscale domains in the PM to PI phase, resulting in an identical gradual resistance change for all piezoresistors, regardless of their orientation with respect to the applied strain, thereby manifesting isotropic piezoresistivity. The findings prompt further inquiry into the existence of nanoscale PM-PI phase coexistence and the reversibility of local phase transitions under applied strain in Cr-doped V_2_O_3_ TFs, warranting a more nuanced exploration of the material’s piezoresistive behavior and phase dynamics.

### GF

The GF is the most important material properties for a successful piezoresistive material. Substituting doped silicon with a material with a higher GF results in a direct improvement of the sensor performance such as signal-to-noise ratio and the resolution of the sensor. More importantly it allows the designer to trade-off this increased GF for other sensor improvements such as increased robustness, smaller size, better linearity, reduced influence from package stress, less power and less lifetime drift. For instance, in piezoresistive pressure sensors it allows to design smaller, thicker membranes with smaller strains without loss of sensitivity but increased sensor robustness and decreased sensor size. In thermal-piezoresistive coupled resonators a larger GF enables further Q factor improvement to achieve lower power consumption and better signal-to-noise^28^. Although many materials have been discovered with high GF, the majority is not practical to implement in multiple mass-market MEMS sensors applications and does not allow the trade-off of higher GF in improved sensor parameters. Materials such as nanotubes and nanowires show very high GF but are very difficult to fabricate in high strain locations with a good mechanical contact^29–32^. Besides high GF materials, also clever new piezoresistive principles were reported to show a high GF. Straining of CMOS circuitry or reducing/increasing connections has some great potential for strain sensing applications, but they are less practical to implement in other MEMS sensors such as pressure sensors or accelerometers^33–35^. Doped silicon owns its successful, widespread use in multiple mass-market piezoresistive sensor applications due to its easy integration and superior chemical, mechanical and electrical properties: good patternability, strong mechanical connection to the substrate, low Ohmic contact to metals and linear resistivity change with strain. To replace doped silicon in several mass-market piezoresistive sensor applications, a material with a high GF is needed that equally excels in these chemical, mechanical and electrical properties. This makes Cr-doped V_2_O_3_ TF potentially an ideal piezoresistive material for the replacement of doped silicon. With the epitaxial bond of Cr-doped V_2_O_3_ TF to the substrate, a strong mechanical connection ensures a good transfer of strain from the substrate to the piezoresistors. Cr-doped V_2_O_3_ TF piezoresistors can easily be patterned and fabricated in high strain locations of the sensor. Due to a low Ohmic contact between with metals, the material can be electrically connected to external circuitry. The linear resistance change with strain and lack of hysteresis allows a sensor with Cr-doped V_2_O_3_ TF piezoresistors to be easily calibrated. The material is fabricated with a MBE system, allowing high-volume manufacturing integration in present-day cleanrooms. In Table 1, the GF_L_ and GF_T_ of Cr-doped V_2_O_3_ TF is compared to doped silicon and other piezoresistive materials that share similar chemical, mechanical and electrical properties that are relevant for implementation in piezoresistive MEMS sensors.

**Table 1:** Longitudinal and transversal gauge factor of different materials

Material	GF longitudinal	GF transversal
Metals used for piezoresistive sensors^2^	2 to 4.8	–
p-type doped Si [110]^23^	*120*	−110
n-type doped Si [100]^23^	*−130*	69
p-type doped Ge^23^	*66*	–
n-type doped Ge^23^	*−103*	93
p-type 6H-SiC^39^	*27*	–
n-type 6H-SiC^39^	22	6
Si_0.9_ Ge_0.1_^21^	99	125
V-doped MoS_2_^40^	*140*	–
4% Cr-doped V_2_O_3_ thin film [this work]	222	217

### Metal-insulating phase transition with strain

Because of the huge difference in conductivity between the metal phase and insulating phase, a strain induced phase transition hold great potential for new piezoresistive materials. In this work is demonstrated that a Mott metal-insulator transition at room temperature can be induced with external strain and that this new piezoresistive transduction principle shows promising potential for successful integration in MEMS sensors. Although the fabrication of Cr-doped V_2_O_3_ TF is currently only possible on a sapphire substrate, limiting the integration of Cr-doped V_2_O_3_ TF within the MEMS fabrication process, mature technologies such as silicon-on-sapphire pressure sensor technologies provide feasible pathways for the seamless application of Cr-doped V_2_O_3_ TF in MEMS sensors^36^. Additionally, many other materials have a phase diagram with potentially an interesting metal-insulator transition phase transition with strain. Further research and development in this area holds great promise for enhancing the performance and capabilities of MEMS pressure sensors, accelerometer, mass flow sensor and resonators^37,38^.

## Conclusion

In this study, the piezoresistivity of Cr-doped V_2_O_3_ thin film was examined through the application of four-point bending to strain the material. With a GF_L_ of 222, Cr-doped V_2_O_3_ thin film piezoresistors exhibit a large resistivity change with strain. This substantial piezoresistivity is attributed to local MIT metal-insulating transitions induced by strain, leading to an overall gradual phase transition of the piezoresistor. Furthermore, isotropic piezoresistivity coefficients were discovered for Cr-doped V_2_O_3_ thin film, resulting in a significant orientation-independent resistance change of Cr-doped V_2_O_3_ thin film piezoresistors with strain. This unique property presents promising opportunities for the utilization of this material in various applications and sensors. To demonstrate the potential of this new piezoresistive material in MEMS sensor applications, a sapphire pressure sensor with Cr-doped V_2_O_3_ thin film piezoresistors was fabricated. For a 4.2 mm large sapphire membrane with a thickness of 98 μm, a sensitivity of 21.81 mV/V/bar was measured at 20 °C. The investigation into the piezoresistivity of Cr-doped V_2_O_3_ thin film has revealed that the Mott metal-insulator transition can be induced with strain. This remarkable new piezoresistive transduction principle holds great potential for use of Cr-doped V_2_O_3_ thin film, or other materials with a Mott metal-insulator transition, in MEMS sensors ultimately contributing to advancements in various fields such as healthcare, automotive, aerospace and beyond. Additionally, the unique combination of large resistivity change with strain and isotropic piezoresistivity coefficients make this material a promising candidate for new sensor applications.

## Supplementary information

Supplementary material Study on the piezoresistivity of Cr-doped V_2 O_3 thin film for MEMS sensor applications
