# Supplementary material for: Study on the piezoresistivity of Cr-doped V2O3 thin film for MEMS sensor applications
Source: Microsyst Nanoeng. 2024 Dec 14;10:189. doi: 10.1038/s41378-024-00807-0 (PMC11645411; doi:10.1038/s41378-024-00807-0)
Supplement: Supplementary file 1 — Supplementary material Study on the piezoresistivity of Cr-doped V_2 O_3 thin film for MEMS sensor applications [file 41378_2024_807_MOESM1_ESM.docx]

Nature

Supplementary Material for

Study on the piezoresistivity of Cr-doped $V_{2}O_{3}$ thin film for MEMS sensor applications

In the format provided by the authors and unedited

Michiel Gidts^1^*, Wei-Fan Hsu^2^, Maria Recaman Payo^2^, Shaswat Kushwaha^3,4^, Frederik Ceyssens^1,5^, Dominiek Reynaerts^3^, Jean-Pierre Locquet^2^, Michael Kraft^1^ and Chen Wang^1^*

^1^ Micro- and Nanosystems, Department of Electrical Engineering, KU Leuven, Kasteelpark Arenberg 10, 3001 Leuven, Belgium;

^2^ Functional Oxides Coating Center, Department of Physics and Astronomy, KU Leuven, Celestijnenlaan 200D, 3001 Leuven, Belgium;

^3^ Manufacturing Processes and Systems, Department of Mechanical Engineering, KU Leuven, Celestijnenlaan 300, 3001 Leuven, Belgium

^4^ Flanders Make, Gaston Geenslaan 8, 3001 Leuven, Belgium

^5^ Membraanscheidingen, Adsorptie, Katalyse en Spectroscopie voor Duurzame Oplossingen, Department of Microbial and Molecular Systems, KU Leuven, Kasteelpark Arenberg 22, 3001 Leuven, Belgium;

***** Correspondance: [michiel.gidts@gmail.com](mailto:michiel.gidts@gmail.com); [chenwang767@163.com](mailto:chenwang767@163.com);

Email of authors: Michiel Gidts (michiel.gidts@gmail.com); Wei-Fan Hsu (peter.hsu@kuleuven.be); Maria Recaman Payo (maria.recamanpayo@kuleuven.be); Shaswat Kushwaha (shashwat.kushwaha@kuleuven.be); Frederik Ceyssens (frederik.ceyssens@kuleuven.be); Dominiek Reynaerts (dominiek.reynaerts@kuleuven.be); Jean-Pierre Locquet (jeanpierre.locquet@kuleuven.be); Michael Kraft (michael.kraft@kuleuven.be); Chen Wang (chenwang767@163.com)

**Table of Contents:**

**2**

1. **Axis system**

**2**

1. **Derivation of** $\boldsymbol{\pi}_{\boldsymbol{L}}$**,** $\boldsymbol{\pi}_{\boldsymbol{T}}$ **and** $\boldsymbol{\pi}_{\boldsymbol{S}}$ **of Cr-doped** $\boldsymbol{V}_{\boldsymbol{2}}\boldsymbol{O}_{\boldsymbol{3}}$ **thin film**

**3**

1. **Four-point bending test setup and simulation**

**4**

1. **Pressure sensor measurement setup**

1. Axis system

In Figure S1, the axis systems used in the paper is shown. The x-axis of the resistor axis system coincides with the long direction of the piezoresistor. The x-axis of the stress axis system coincides with the long direction of the beam sample.


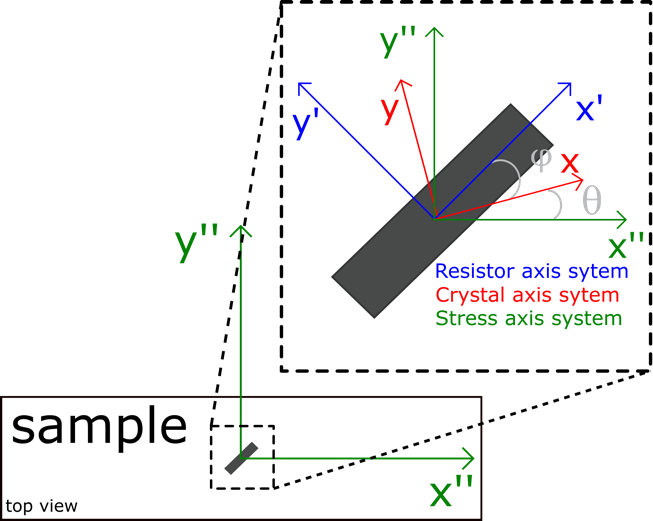


**Fig. S1 Axis system.** Schematic of resistor on sample and its in-plane rotation with respect to the crystal axis system and stress axis system.

2. Derivation of $\boldsymbol{\pi}_{\boldsymbol{L}}$, $\boldsymbol{\pi}_{\boldsymbol{T}}$ and $\boldsymbol{\pi}_{\boldsymbol{S}}$ of Cr-doped $\boldsymbol{V}_{\boldsymbol{2}}\boldsymbol{O}_{\boldsymbol{3}}$ thin film

The piezoresistivity coefficient tensor in the crystal axis system is given by [20]:

|  | $\pi_{Cr-V_{2}O_{3}}=\left[ \begin{matrix} \pi_{11} & \pi_{12} & \pi_{13} & \pi_{14} & 0 & 0 \\ \pi_{12} & \pi_{11} & \pi_{13} & {-\pi}_{14} & 0 & 0 \\ \pi_{31} & \pi_{31} & \pi_{33} & 0 & 0 & 0 \\ \pi_{41} & -\pi_{41} & 0 & \pi_{44} & 0 & 0 \\ 0 & 0 & 0 & 0 & \pi_{44} & 2\pi_{41} \\ 0 & 0 & 0 & 0 & \pi_{14} & \pi_{11}-\pi_{12} \end{matrix} \right]$ | (1) |
| --- | --- | --- |

A rotation of the piezoresistivity coefficient tensor to the resistor axis system is given by [41]:

|  | $\pi_{Cr-V_{2}O_{3}}^{'}=\left[ \begin{matrix} \pi_{11}^{'} & \pi_{12}^{'} & \pi_{13}^{'} & \pi_{14}^{'} & \pi_{15}^{'} & \pi_{16}^{'} \\ \pi_{21}^{'} & \pi_{22}^{'} & \pi_{23}^{'} & \pi_{24}^{'} & \pi_{25}^{'} & \pi_{26}^{'} \\ \pi_{31}^{'} & \pi_{32}^{'} & \pi_{33}^{'} & \pi_{34}^{'} & \pi_{35}^{'} & \pi_{36}^{'} \\ \pi_{41}^{'} & \pi_{42}^{'} & \pi_{43}^{'} & \pi_{44}^{'} & \pi_{45}^{'} & \pi_{46}^{'} \\ \pi_{51}^{'} & \pi_{52}^{'} & \pi_{53}^{'} & \pi_{54}^{'} & \pi_{55}^{'} & \pi_{56}^{'} \\ \pi_{61}^{'} & \pi_{62}^{'} & \pi_{63}^{'} & \pi_{64}^{'} & \pi_{65}^{'} & \pi_{66}^{'} \end{matrix} \right]=T\pi_{Cr-V_{2}O_{3}}T^{-1}$ | (1) |
| --- | --- | --- |

With the transformation matrix t from the crystal axis system to the resistor axis system given by:

|  | $t=\left( \begin{matrix} l_{1} & m_{1} & n_{1} \\ l_{2} & m_{2} & n_{2} \\ l_{3} & m_{3} & n_{3} \end{matrix} \right)=\left( \begin{matrix} \cos(\varphi) & \sin(\varphi) & 0 \\ -\sin(\varphi) & \cos(\varphi) & 0 \\ 0 & 0 & 1 \end{matrix} \right)$ | (1) |
| --- | --- | --- |

T is derived from the transformation matrix t:

|  | $T= \left( \begin{matrix} \begin{matrix} l_{1}^{2} & m_{1}^{2} & n_{1}^{2} \\ l_{2}^{2} & m_{2}^{2} & n_{2}^{2} \\ l_{3}^{2} & m_{3}^{2} & n_{3}^{2} \end{matrix} & \begin{matrix} 2m_{1}n_{1} & 2n_{1}l_{1} & 2l_{1}m_{1} \\ 2m_{2}n_{2} & 2n_{2}l_{2} & 2l_{2}m_{2} \\ 2m_{3}n_{3} & 2n_{3}l_{3} & 2l_{3}m_{3} \end{matrix} \\ \begin{matrix} l_{2}l_{3} & m_{2}m_{3} & n_{2}n_{3} \\ l_{3}l_{1} & m_{3}m_{1} & n_{3}n_{1} \\ l_{1}l_{2} & m_{1}m_{2} & n_{1}n_{2} \end{matrix} & \begin{matrix} m_{2}n_{3}+m_{3}n_{2} & n_{2}l_{3}+n_{3}l_{2} & m_{2}l_{3}+m_{3}l_{2} \\ m_{3}n_{1}+m_{1}n_{3} & n_{3}l_{1}+n_{1}l_{3} & m_{3}l_{1}+m_{1}l_{3} \\ m_{1}n_{2}+m_{2}n_{1} & n_{1}l_{2}+n_{2}l_{1} & m_{1}l_{2}+m_{2}l_{1} \end{matrix} \end{matrix} \right)$  $= \left( \begin{matrix} \begin{matrix} \cos^{2} \left( \varphi\right) & \sin^{2} \left( \varphi\right) & 0 \\ \sin^{2} \left( \varphi\right) & \cos^{2} \left( \varphi\right) & 0 \\ 0 & 0 & 1 \end{matrix} & \begin{matrix} 0 & 0 & \sin(2\varphi) \\ 0 & 0 & -\sin(2\varphi) \\ 0 & 0 & 0 \end{matrix} \\ \begin{matrix} 0 & 0 & 0 \\ 0 & 0 & 0 \\ -\sin\left( 2\varphi\right)/2 & \sin\left( 2\varphi\right)/2 & 0 \end{matrix} & \begin{matrix} \cos(\varphi) & -sin (\varphi) & 0 \\ \sin(\varphi) & \cos(\varphi) & 0 \\ 0 & 0 & \cos(2\varphi) \end{matrix} \end{matrix} \right)$ | (1) |
| --- | --- | --- |

and $T^{-1}$ is than given by:

|  | $T^{-1}=\left( \begin{matrix} \begin{matrix} l_{1}^{2} & l_{2}^{2} & l_{3}^{2} \\ m_{1}^{2} & m_{2}^{2} & m_{3}^{2} \\ n_{1}^{2} & n_{2}^{2} & n_{3}^{2} \end{matrix} & \begin{matrix} 2l_{2}l_{3} & 2l_{3}l_{1} & 2l_{1}l_{2} \\ 2m_{2}m_{3} & 2m_{3}m_{1} & 2m_{1}m_{2} \\ 2n_{2}n_{3} & 2n_{3}n_{1} & 2n_{1}n_{2} \end{matrix} \\ \begin{matrix} m_{1}n_{1} & m_{2}n_{2} & m_{3}n_{3} \\ n_{1}l_{1} & n_{2}l_{2} & n_{3}l_{3} \\ l_{1}m_{1} & l_{2}m_{2} & l_{3}m_{3} \end{matrix} & \begin{matrix} m_{2}n_{3}+m_{3}n_{2} & m_{3}n_{1}+m_{1}n_{3} & m_{1}n_{2}+m_{2}n_{1} \\ n_{2}l_{3}+n_{3}l_{2} & n_{3}l_{1}+n_{1}l_{3} & n_{1}l_{2}+n_{2}l_{1} \\ m_{2}l_{3}+m_{3}l_{2} & m_{3}l_{1}+m_{1}l_{3} & m_{1}l_{2}+m_{2}l_{1} \end{matrix} \end{matrix} \right)$  $=\left( \begin{matrix} \begin{matrix} \cos^{2} \left( \varphi\right) & \sin^{2} \left( \varphi\right) & 0 \\ \sin^{2} \left( \varphi\right) & \cos^{2} \left( \varphi\right) & 0 \\ 0 & 0 & 1 \end{matrix} & \begin{matrix} 0 & 0 & -sin (2\varphi) \\ 0 & 0 & \sin(2\varphi) \\ 0 & 0 & 0 \end{matrix} \\ \begin{matrix} 0 & 0 & 0 \\ 0 & 0 & 0 \\ \sin\left( 2\varphi\right)/2 & -\sin\left( 2\varphi\right)/2 & 0 \end{matrix} & \begin{matrix} \cos(\varphi) & \sin(\varphi) & 0 \\ -sin (\varphi) & \cos(\varphi) & 0 \\ 0 & 0 & \cos(2\varphi) \end{matrix} \end{matrix} \right)$ | (1) |
| --- | --- | --- |

For a piezoresistor rotated to the resistor axis system as shown in Figure S1, the piezoresistivity coefficient $\pi_{L}$, $\pi_{T}$ and $\pi_{S}$ in the resistor axis system can be expressed as:

|  | $\pi_{L}=\pi_{11}^{'}=\left( \cos^{2} (\varphi\right)\pi_{11}+\sin^{2} \left( \varphi\right)\pi_{12})\cos^{2} \left( \varphi\right)$ $+ \left( \cos^{2} (\varphi\right)\pi_{12}+\sin^{2} \left( \varphi\right)\pi_{11})\sin^{2} \left( \varphi\right)$ $+ \left( \pi_{11}-\pi_{12} \right)\sin^{2} (2\varphi)/2$ $=\pi_{11}$ | (2) |
| --- | --- | --- |
|  | $\pi_{T}=\pi_{12}^{'}=\left( \cos^{2} (\varphi\right)\pi_{12}+\sin^{2} \left( \varphi\right)\pi_{11})\cos^{2} \left( \varphi\right)$ $+ \left( \cos^{2} (\varphi\right)\pi_{11}+\sin^{2} \left( \varphi\right)\pi_{12})\sin^{2} \left( \varphi\right)$ $- \left( \pi_{11}-\pi_{12} \right)\sin^{2} (2\varphi)/2$ $=\pi_{12}$ | (3) |
|  | $\pi_{S}=\pi_{16}^{'}=(-\sin\left( 2\varphi\right)\pi_{11}+\sin\left( 2\varphi\right)\pi_{12})\cos^{2} \left( \varphi\right)$ $+ (-\sin\left( 2\varphi\right)\pi_{12}+\sin\left( 2\varphi\right)\pi_{11})\sin^{2} \left( \varphi\right)$ $+ \left( \pi_{11}-\pi_{12} \right)\cos(2\varphi)\sin\left( 2\varphi\right)$ $=0$ | (4) |

Since the piezoresistivity coefficient $\pi_{L}$, $\pi_{T}$ and $\pi_{S}$ are independent of the angle $\varphi$, the resistance change of a Cr-doped $V_{2}O_{3}$ TF piezoresistor with stress rotated in the XY-plane of the crystallographic framework is independent of the rotation angle $\varphi$.

3. Four-point bending test setup and simulation

A four-point bending test setup was constructed of an aluminum framework with components produced via three-dimensional printing and various electrical elements. A 3D-printed base beam, characterized by its rigidity, was securely affixed to the aluminum structure using a fastener. In order to induce an upward deflection in the sample, resulting in tensile strain within the piezoresistive elements, a beam configuration with a 10 mm inter-beam spacing was utilized at the base. Subsequently, the sample was positioned atop these beams, followed by the application of another beam assembly, this time with a 20 mm separation between beams, placed directly on top of the sample (see Figure S2). Precision in the alignment of the sample and the beam assemblies, both at the bottom and top, was meticulously maintained. For scenarios requiring a downward flexure of the sample, which invokes a compressive stress in the piezoresistors, the positioning of the beam assemblies was reversed. It is noteworthy that the innovative design of the sample holder ensures that the sample is exclusively supported by the beams when installed within the apparatus. The beams in question were fabricated using stereolithography 3D printing, achieving a resolution of 50 μm, and were composed of an insulating material possessing considerable stiffness (Young's modulus of 2.6 GPa). To facilitate the transmission of electrical signals to and from the sample, wire bonding was employed to connect the sample to printed circuit boards (PCBs) situated on either side. The application of force to the sample was methodically increased by adding weights onto the upper 3D-printed beam structure, as depicted in Figure S2a. To quantify the strain elicited in the sample by this applied force, simulations were conducted using the COMSOL Multiphysics software, see Figure S3.

**
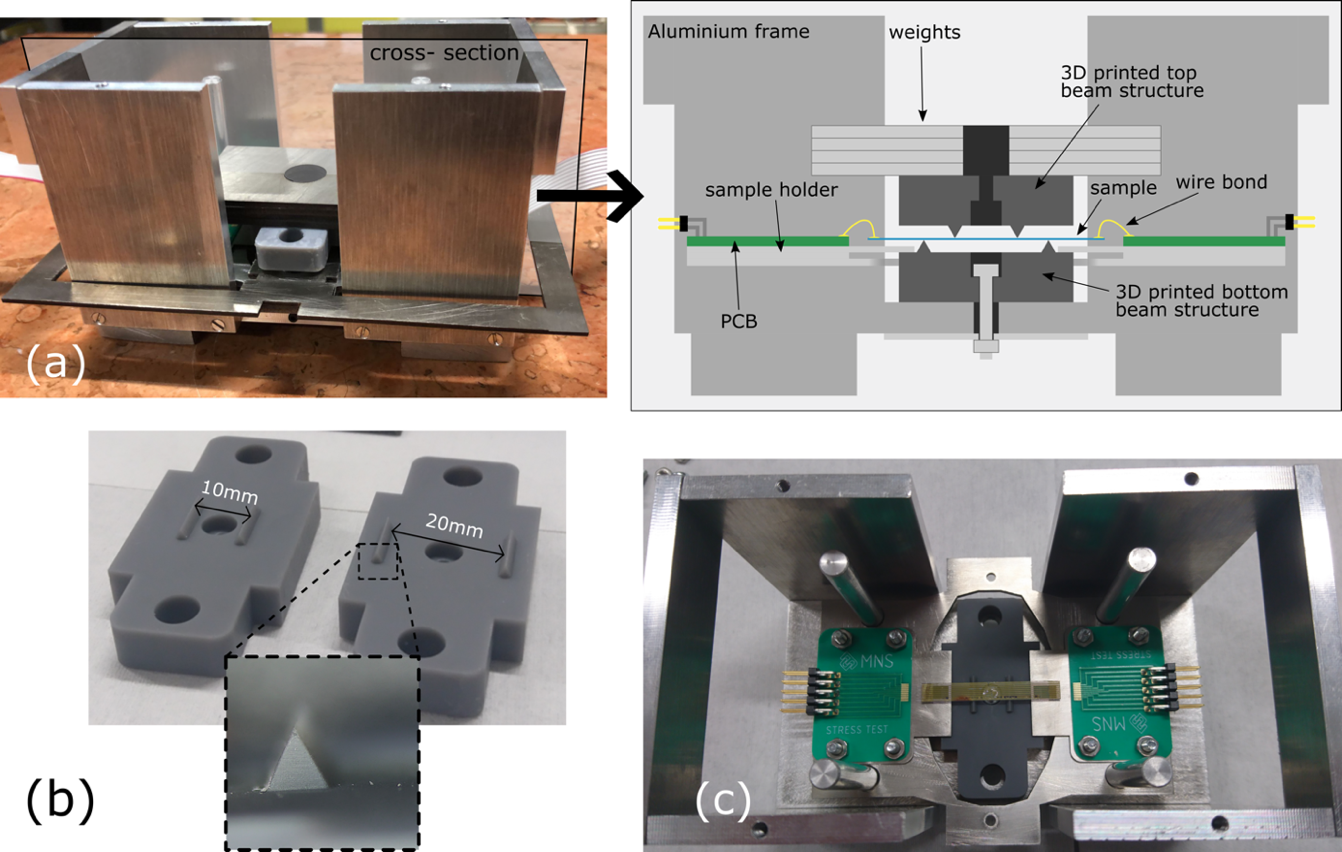
**

**Fig. S2 Four-point bending test setup.** (a) Four-point bending test setup with sample being bend by weights. (b) 3D printed beam structures that support the sample and cause bending of the sample. (c) Test sample on 3D printed bottom beam structure in test setup.

**
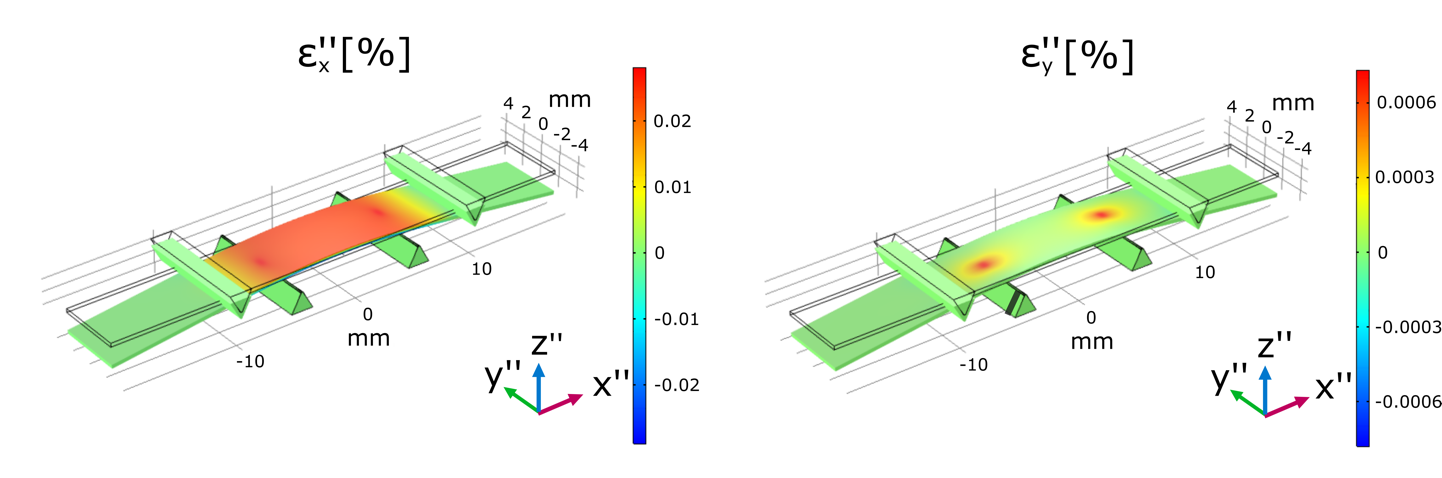
**

**Fig. S3 Four-point bending simulation.** COMSOL simulation of strain in sapphire sample with a force of 3 N on the top beams.

4. Pressure sensor measurement setup

The pressure sensor measurement setup composed of a precision reference pressure sensor, a personal computer (PC), a source meter, a National Instruments (NI) data acquisition card, a temperature gauge and a pressurized chamber (see Figure S4). The printed circuit board (PCB), on which the pressure sensor was glued, was interfaced through HDMI cables to an ancillary circuit. The PCB featured multiple apertures for affixing screws from the pressurized chamber, ensuring a secure connection. Constructed from aluminium, the chamber encapsulated the sensor die, ensuring a hermetic seal against external environments through the employment of multiple O-rings. Temperature regulation was achieved by situating the chamber beneath a thermostream unit, which provided a stream of clean, dry air essential for precise thermal testing of electronic components. Utilizing LabView software, the testing parameters were meticulously programmed, enabling the systematic incrementation of differential pressure from 0 to 1.8 bar in 0.1 bar increments at each designated temperature, with the procedure being replicated multiple times to ensure reliability.


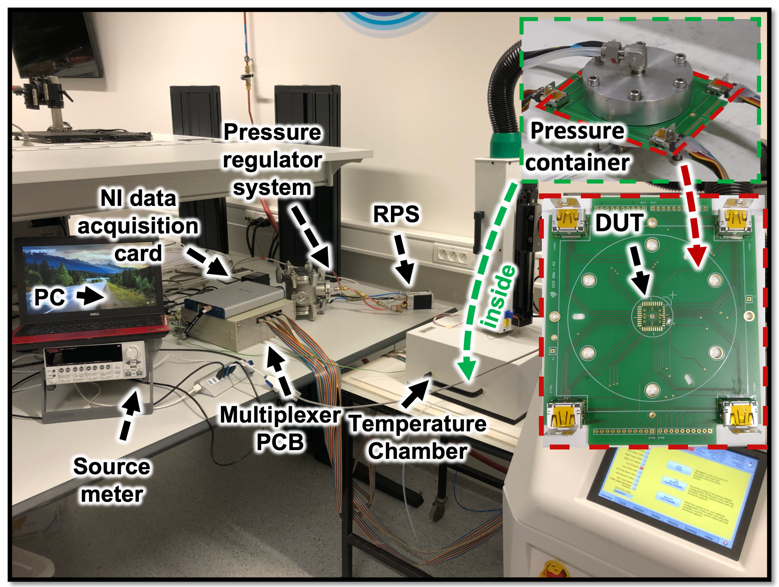


**Fig. S4 Pressure sensor measurement setup.** Setup used to characterize the Cr-doped $V_{2}O_{3}$ TF sapphire pressure sensor.
